# Comments on: “Transcranial Direct Current Stimulation for Obsessive-Compulsive Disorder: A Systematic Review”

**DOI:** 10.3390/brainsci8040055

**Published:** 2018-03-27

**Authors:** Mohammad Alwardat, Mohammad Etoom

**Affiliations:** 1 School of Neuroscience, Faculty of Medicine and Surgery, University of Rome “Tor Vergata”, Via Montpellier 1, 00133 Rome, Italy; 2 Physical Therapy Department, Al-Isra University, P.O. Box 33, Amman 11622, Jordan; mohammed.etoom@yahoo.com

Dear Editor, Brunelin et al. [1] recently conducted a systematic review that evaluated the effect of applied transcranial direct current stimulation (tDCS) on patients with obsessive compulsive disorder (OCD). It is encouraging to see more safety results concerning repeated sessions of tDCS to improve cognitive functioning in various psychiatric conditions. We have several comments regarding this review.

The review is reported according to the Preferred Reporting Items for Systematic Review and Meta-Analysis (PRISMA) format, but the inclusion criteria are ambiguous. The authors do not sufficiently follow the PICO format (P: participants, I: intervention, C: comparison, O: outcomes). The authors state the inclusion criteria as: “(i) full length original articles published in English language in peer-reviewed journals; (ii) patients with OCD according to DSM or ICD-10 criteria; (iii) detailed description of the stimulation method; and (iv) the use of repeated sessions of tDCS”.

Additionally, the authors state the review aims to: “provide a comprehensive overview of existing literature on the effects of tDCS applied as a therapeutic tool to reduce OC symptoms in patients with treatment-resistant OCD and to discuss future applications of tDCS in OCD”. Surprisingly, the review did not clearly report the other components of inclusion criteria like comparison and outcome measures. Running a systematic review without full knowledge about the inclusion criteria can lead to problems with assessing the validity, applicability, and comprehensiveness of the systematic review [2].

The authors non-systematically employed articles with wide and heterogeneous methodological designs such as randomized control trials and observational studies but there was no clear selection criteria regarding the methodological designs of the studies included. Furthermore, the qualities of evidence, risk of bias and heterogeneity of the included studies have not been reported. The authors have ignored other major components of a full systematic review. The aim of the systematic review is to assess the quality of included articles and heterogeneity to disclose the risk of bias and conclude the level of evidence. The concluded level of evidence is a major part to provide the research and clinical recommendations. Moreover, the systematic reviews have to provide the effectiveness of interventions with the level or quality of included evidence [2,3]. Thus, to determine the level of evidence, we believe that adapting the Grading of Recommendations Assessment, Development and Evaluation GRADE approach is highly recommended and efficient.

The systematic review is different from other types of literature reviews. It must provide an explicit, reproducible methodology and include a systematic search that attempts to identify all studies that would meet the eligibility criteria [3,4]. This unique construction requires the Methods section of a systematic review to be evaluated much like a quantitative research study. However, this review has also several troubling flaws in the methods. The authors reported using PubMed, there was also the opportunity to use Medical Subject Headings (MeSH) in the search. Using subject headings in addition to keywords is a key point of searching for studies according to Cochrane Handbook for Systematic Reviews of Interventions [4].
